# Breakdown of Continuum Fracture Mechanics at the Nanoscale

**DOI:** 10.1038/srep08596

**Published:** 2015-02-26

**Authors:** Takahiro Shimada, Kenji Ouchi, Yuu Chihara, Takayuki Kitamura

**Affiliations:** 1 Department of Mechanical Engineering and Science, Kyoto University, Kyoto 615-8540, Japan

## Abstract

Materials fail by the nucleation and propagation of a crack, the critical condition of which is quantitatively described by fracture mechanics that uses an intensity of singular stress field characteristically formed near the crack-tip. However, the continuum assumption basing fracture mechanics obscures the prediction of failure of materials at the nanoscale due to discreteness of atoms. Here, we demonstrate the ultimate dimensional limit of fracture mechanics at the nanoscale, where only a small number of atoms are included in a singular field of continuum stress formed near a crack tip. Surprisingly, a singular stress field of only several nanometers still governs fracture as successfully as that at the macroscale, whereas both the stress intensity factor and the energy release rate fail to describe fracture below a critically confined singular field of 2–3 nm, i.e., breakdown of fracture mechanics within the framework of the continuum theory. We further propose an energy-based theory that explicitly accounts for the discrete nature of atoms, and demonstrate that our theory not only successfully describes fracture even below the critical size but also seamlessly connects the atomic to macroscales. It thus provides a more universal fracture criterion, and novel atomistic insights into fracture.

Understanding the nature of fracture, a catastrophic failure of materials, remains a major challenge in a wide range of fields including engineering, physics, materials science, biology, and geophysics, because fracture is both a physically essential phenomenon and a practically inevitable issue that all materials in all range of scales intrinsically possess and commonly suffer from Refs. 1,2,3,4,5,6,7,8,9,10,11,12,13. The fracture of materials is generally initiated locally from a crack tip, which leads to a global failure of materials through crack propagation across the entire structure. Therefore, the mechanical behavior of crack is of central importance to the fracture. Fracture mechanics^1^, established on the basis of the continuum mechanics theory, provides a theoretical framework to describe the critical conditions at which a crack becomes mechanically unstable and begins to propagate. The presence of a crack significantly concentrates mechanical stress or strain to the immediate vicinity of the crack-tip and the stress diverges as a singularity of 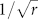 at the tip^14^, which intensively accumulates strain energy to the crack-tip area as the driving force to initiate fracture. Therefore, not the maximum stress at a single point, but the stress intensity of this continuum “singular field” near the crack tip determines the initiation of fracture. A huge number of studies have been conducted over a long period based on this fracture mechanics concept, both experimentally and theoretically for a wide range of specimen sizes, from meters to micrometers^1^,^2^,^3^,^4^,^5^,^6^,^7^,^8^,^9^,^10^,^11^,^12^,^13^,^14^,^15^,^16^. These studies have demonstrated that fracture is successfully described by the singular field of continuum stress, regardless of the size of materials, nevertheless fracture is ultimately characterized by discrete events at the atomic scale, such as bond breaking^16^.

However, ambiguity emerges at the nanoscale: As the structural dimensions of materials are further scaled down to nanometers, the singular stress field formed near the crack tip is similarly confined to nanometers, where only an extremely smaller number of atoms are present with respect to the macroscale materials. This situation is clearly inconsistent with the facture mechanics concept based on the continuum theory that postulates the presence of a sufficiently large number of atoms to regard even a crack-tip area as continuum media. This inconsistency brings fundamental questions of to what scale does fracture mechanics go and what is alternative principle governing fracture below a critical dimension at which fracture mechanics breaks down. Although very few attempts toward this critical issue have been done due to experimental difficulties at the nanometer scale, a result suggested that even a singular stress field of several tens of nanometers would still govern fracture^2^,^17^. Direct fracture tests for even smaller specimens, however, are experimentally almost intractable. Thereby, interpretation from the continuum (fracture) mechanics perspective and finding the limit still remains a considerable and hard challenge.

This Letter provides striking results that fracture mechanics based on the continuum theory still successfully describe fracture even in an extremely confined singular stress field of only several nanometers, according to ideal fracture experiments for a brittle material *in silico*. In addition, we first capture the breakdown of fracture mechanics and successfully identify the lower applicable limit (dimensional bound) for fracture mechanics. An attempt is also made to consistently describe fracture even below the lower limit by a straightforward extension of the fracture mechanics concept to the atomic scale.

Fracture tests are carefully performed *in silico* for pre-cracked nanoscale specimens of a brittle material, where the atomistic nature of fracture is particularly clear enough to determine. Geometry of our nanoscale specimens is shown in Fig. 1a. The crack length 2*a* is one third of the plate width, 2*W*(*a* = *W*/3). Based on Saint-Venant's principle, the plate height *H* is set to be large enough (*H* = 8*W*) to avoid undesirable effects from the loading points to the stress field near the crack. Several tens of specimens are prepared with the same geometry and different sizes, ranging from 2*W* = 2.1 to 276 nm, which includes up to 4,147,200 atoms. The samples consist of a single crystal of a silicon diamond-cubic structure, where the 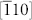, 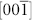, and [110] axes correspond to the *x*, *y*, and *z* directions of the specimens, respectively. The crack is thus along the (110) cleavage plane with the [001] crack front. The brittle nature of fracture at the crack tip can be described by a bond-order potential of modified Stillinger-Weber form^18^, which gives the lattice constant of 5.431 nm and the mode I fracture toughness for the present crack system (110)[001] in the bulk of 1.03 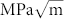, and is in excellent agreement with the experimental values of 5.431 nm^19^ and 0.90–1.15 
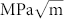
20,21,22, respectively. We apply constant loads to the top and bottom of specimens as precondition for the present fracture tests. A quasi-static tensile test is performed by applying a stepwise increment of load *P* to the atoms at the top and bottom of the specimen. At each loading step, the atomic structure is fully relaxed until all forces acting on the atoms are less than 1.0 × 10^−9^ nN. We carefully tested around the critical load where the crack begins to propagate with a much smaller increment of load (less than 0.05% of the critical load). During the tensile test, the *y* cell dimension is fixed with a periodical boundary, i.e., a plane-strain condition. In addition to tensile testing, a quasi-static bending test is performed for half-sized specimens with an edge crack, as shown in the bottom panel of Fig. 1(a). The bending test achieves the same (mode I) stress field near the crack tip while the far stress field is different from that of the tensile test. To carefully cross-check the results of above classical atomic simulations, we additionally perform first-principles density-functional theory calculations for crack-propagation using the commonly-used supercell set-up^16^. The detailed simulation models and procedure are shown in Supplementary Material.

During the tensile tests, the displacement *d* at the loading point linearly increases with the applied load *P* (Fig. 1(b)). Subsequently, the displacement increases abruptly when the applied load reaches the critical value *P_c_*, where the crack becomes mechanically unstable and begins to propagate along the (110) cleavage plane (Fig. 1c). The global deformation is thus linearly elastic and the subsequent fracture is purely brittle. Such brittle nature of fracture is consistently observed in all specimens tested under both tension and bending. The results of fracture tests are investigated from a continuum-mechanics perspective by assuming the specimen to be an elastic continuum medium in order to explore the lower size limit of fracture mechanics^15^. In a specimen of *W* = 104 nm, the stress intensively concentrates near the crack-tip and forms a singular field inversely proportional to the square root of distance, 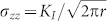, where *K_I_* denotes the stress intensity factor (SIF) (Fig. 2(a)). The critical SIF at fracture is evaluated to be 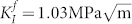, which is in perfect agreement with the fracture toughness 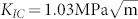. This evidently indicates that the fracture mechanics criterion, i.e., the crack propagates just when the SIF reaches the fracture toughness, is applicable. This is also true for the bending test despite of different far stress field. This indicates that fracture is well-dominated by only the singular stress field near the crack tip, the size of which Λ*_K_* = 5.1 and 4.1 nm (*K*-dominant region) for the tensile and bending tests, respectively. On the other hand, in a smaller specimen of *W* = 16 nm, where the *K*-dominant region is Λ*_K_* = 0.8 nm (Fig. 2(b)), fracture contrastingly occurs at 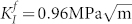. This clearly deviates from the fracture toughness. Here, it should be noted that the result does *not* mean that the crack in the nanoscale specimen becomes mechanically weak (the fracture toughness itself decreases), because the bending test for the same size of specimen gives a contrastingly higher critical SIF of 1.07 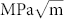. Such deviation is also experimentally observed very recently in graphene with an extremely short crack of 33 nm^2^. Therefore, fracture in such nanoscale specimens is no longer governed by SIF, i.e., continuum fracture mechanics breaks down. The critical size of *K*-dominant region is evaluated to be 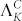 = 2–3 nm (*W* = 40–60 nm in specimen size), as shown in Fig. 2(c) and confirmed by quantum-mechanical tests (Supplementary Fig. S1).

To provide physical insight in depth into the lower limit of fracture mechanics 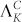, here we investigate the atomistic nature of brittle fracture using our instability mode analysis (see Supplementary Material)^23^, which allows to rigorously capture the deformation mode of atoms at the onset of brittle fracture, and the mode is visualized in Fig. 3(a). The mode clearly shows the behavior of atoms that opens the crack and advances the crack-tip along the (110) cleavage plane through a bond break, and the same fracture mode is consistently observed in all different size of specimens tested. In addition, the analysis based on density-functional theory calculations also gives the same fracture mode (Supplementary Fig. S2). These features evidently represent an intrinsic mode of brittle fracture for the Si crack. The discrete motion of atoms is highly concentrated near the crack-tip, and the size of this fracture-dominant zone Λ*_f_* is estimated to be 0.4–0.6 nm. Here, let us mention a hypothesis of fracture mechanics that the *K*-dominant region must be geometrically large enough compared to a fracture process zone, which includes various nonlinear phenomena resulting in inelastic deformation near the crack-tip^1^. Since Λ*_f_* can be regarded as an atomic-level process zone for brittle fracture, 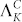 is expected to be related to Λ*_f_*. As shown in Fig. 3(b), fracture mechanics is valid in a large specimen of *W* = 104 nm, where Λ*_K_* is ten times larger than Λ*_f_* and is thus large enough to satisfy the hypothesis Λ*_K_* ≫ Λ*_f_*. On the other hand, 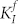 starts to deviate and fracture mechanics breaks down in smaller specimens of *W* = 41 and 16 nm, where Λ*_K_* becomes closer to Λ*_f_* and the hypothesis of fracture mechanics thus breaks down. In fact, the deviation of 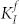 is more dramatically pronounced around Λ*_K_* ≈ Λ*_f_* (Fig. 2(c)). Therefore, Λ*_f_* determines the lower limit of fracture mechanics, roughly estimated to be 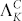 = 3–6Λ*_f_*.

Fracture mechanics provides another criterion of energy release rate (ERR) 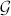, originally proposed by Griffith^24^, and extended by Orowan^25^ and Irwin^26^. The ERR is defined as the released mechanical (strain) energy with infinitesimal change of crack cross-section *A*, and LEFM gives 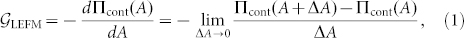
where Π_cont_(*A*) denotes the strain energy of continuum media and *A* is the crack cross-section. In *W* > 60 nm, 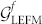 is in good agreement with the fracture toughness 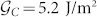 (Fig. 4(a)). However, 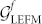 begins to gradually deviate from 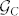 around a specimen size of *W*^C^ = 40–60 nm, i.e., the energy-based LEFM fails. The critical specimen size is *W*^C^ = 40–60 nm, which is consistent with the failure of SIF already discussed because 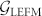 corresponds to SIF with 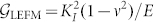, where *E* and 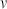 are the Young's modulus and Poisson's ratio, respectively^1^. It should be noted that Pugno and Ruoff proposed quantized fracture mechanics (QFM)^27^, which partially includes the effect of discreteness of atoms at the crack-tip into the continuum fracture mechanics by considering finite advance of crack in continuum media, but the ERR based on QFM still fails to describe fracture below the critical size (Fig. 4(a)). Such failure is because the actual strain energy distribution near the crack-tip is no longer described by continuum assumption in such critically small specimens (Fig. 4(b)). The deviation must be critical for the evaluation of ERR because the strain energy concentrated in the *K*-dominant region predominantly contributes to ERR^1^. Thus, the failure of LEFM and QFM is due to the continuum assumption basing both of them.

Beyond the continuum-based fracture mechanics as discussed above, here we propose a following ERR, where the discreteness of atoms at the crack tip is now fully taken into account by a straightforward extension of the fracture mechanics concept to the atomic scale, as an effective parameter to describe fracture below the lower size limit, 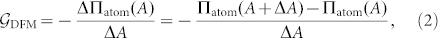
where Π_atom_(*A*) is the potential energy of the simulated atomic specimen with a crack cross-section of *A*. Δ*A* is the finite change of the crack cross-section at the onset of fracture which can be derived from the fracture mode analysis; in the present case it corresponds to a single bond break at the crack-tip as seen in the mode. DFM accounts for the strain energy of discretized atomic body and the discrete nature of atoms at the crack-tip, in contrast to the original ERR which assumes the continuum strain energy and infinitesimal crack advance. In addition, DFM no longer postulates the presence of singular field, suggesting applicability to non-crack systems. Here we call this analytic theory discrete fracture mechanics (DFM), and the critical ERR based on proposed DFM, 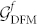, as a function of specimen size *W* is shown in Fig. 4c. The fracture event always occurs when 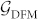 reaches a critical constant value of 5.2 J/m^2^, regardless of the loading conditions, for all specimen sizes, even below *W^c^* = 40–60 nm. The DFM therefore describes the onset of crack propagation successfully even in nanoscale specimens where the continuum fracture mechanics breaks down. In addition, 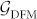 effectively works as a governing parameter, not only at the nanoscale, but also at the macroscale: As the specimen size approaches the macroscale, the finite Δ*A* can be approximately regarded as an infinitesimally small value with respect to the entire size of the specimen (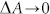) and the strain energy distribution near the crack-tip is well approximated by continuum assumption (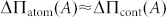; Fig. 4(b)). Thus, 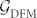 is identical to 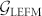 at the macroscale from Eqs. (1) and (2). This seems to be rational because the critical 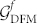 of 5.2 J/m^2^, where fracture occurs, perfectly agrees with the fracture toughness 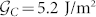, given by the original framework of fracture mechanics. Therefore, DFM consistently and seamlessly bridges the nanometer (atomic) scale and the macroscale (continuum), and successfully describes fracture for all scales.

We have shown that fracture mechanics fails below a critical singular-field size of 2–3 nm. Alternatively, we have proposed a new energy-based theory that now accounts for the discrete nature of atoms, and have demonstrated that it universally describes fracture even below the critical size and provides a seamless connection between the atomic and macroscale (continuum). This success not only contributes to the reliability and design of industrial devices that now consist of nanoscale materials, but also provides additional atomistic insight into fracture toughness, which has simply been considered as materials constants to represent the resistance to fracture within the conventional framework. These results also promote hard experimentation on fracture at the nanoscale and novel theoretical or physical re-interpretation of fracture events of various materials beyond conventional fracture mechanics, leading to new strategies for the improvement of materials strength and toughness.

## Author Contributions

T.S. designed and directed computational experiments, performed first-principles DFT calculations, and wrote the entire manuscript. K.O. and Y.C. performed the atomic simulations, finite element analysis, and atomistic instability analysis. T.K. conceived the project, supervised the work, and provided critical feedback on the manuscript. All authors read and commented on the manuscript.

## Supplementary Material

Supplementary Information Supplementary Figures and Theory

## Figures and Tables

**Figure 1:**
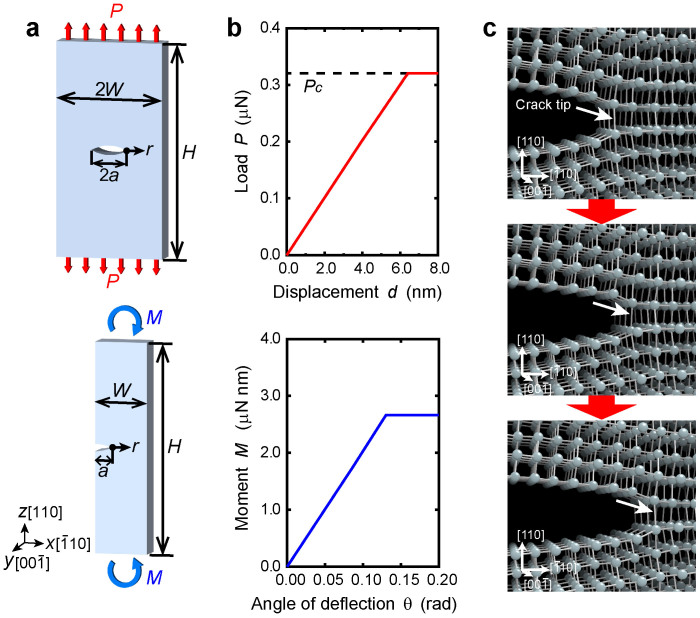
Fracture testing for pre-cracked nanoscale specimens. (a), Geometry and loading conditions of pre-cracked nanoscale specimens for tensile tests (top) and bending tests (bottom). The computational experiments are performed for the different size of specimens of 2*W* = 2.1–276 nm. (b), Tensile load *P* and the corresponding displacement *d* under tensile tests (top), and bending moment *M* and the corresponding angle of deflection *θ* under bending tests (bottom), for a specimen of *W* = 104 nm. (c), Change in atomic configuration during the crack propagation under a critical loading conditions. The crack propagates along the (110) cleavage plane with a sequential bond breaking.

**Figure 2:**
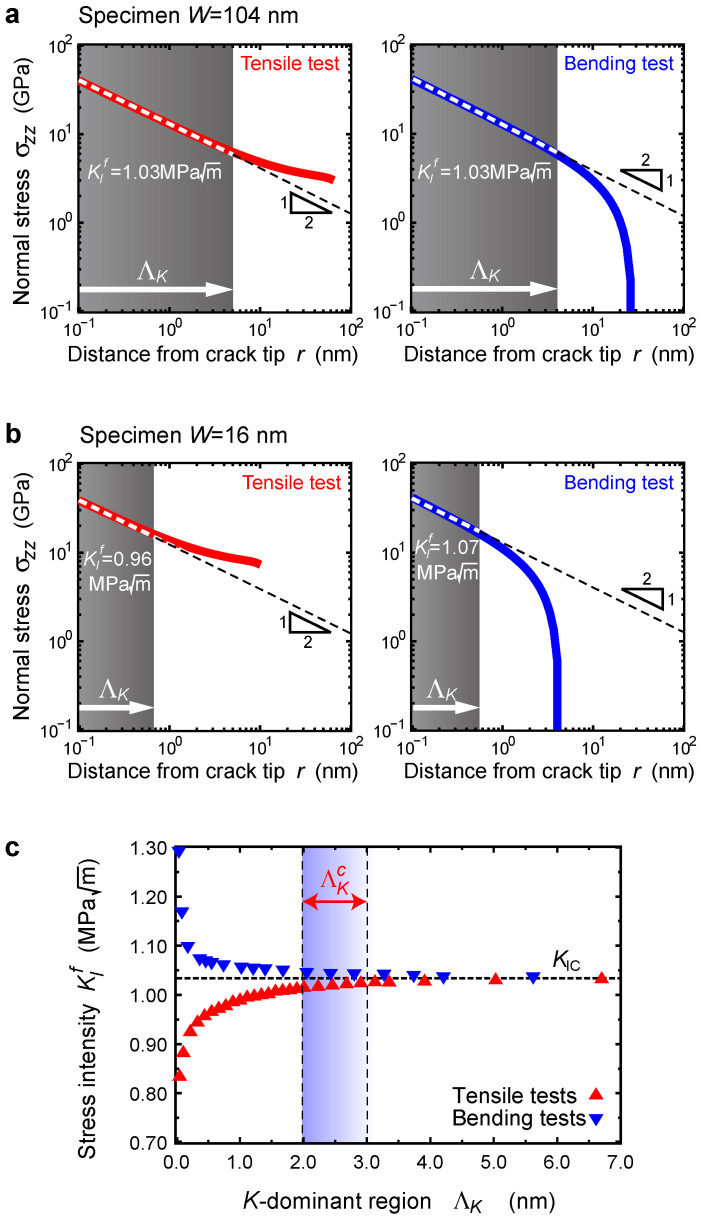
Critical stress intensity of singular field in nanoscale specimens and dimensional limit of continuum fracture mechanics. (a), Normal stress 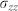 at the onset of fracture as a function of distance from the crack tip *r* along the (110) crack plane under tensile and bending tests (solid red and blue lines, respectively) for a larger specimen of *W* = 104 nm. The dashed lines indicate the slope of a 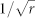 singularity, and the shaded area of Λ*_K_* indicates the *K*-dominant region (singular stress field size) near the crack-tip. (b), Same as Fig. 2a, but for a smaller specimen of *W* = 16 nm. (c), Critical stress intensity factor at fracture 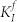 as a function of *K*-dominant region Λ*_K_* obtained by fracture tests for different size of specimens. The horizontal dotted line indicates the fracture toughness *K_IC_* = 1.03 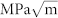 of the corresponding crack system (110)[001]. 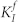 gradually starts to deviate from fracture toughness around the critical size of 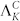 = 2–3 nm.

**Figure 3:**
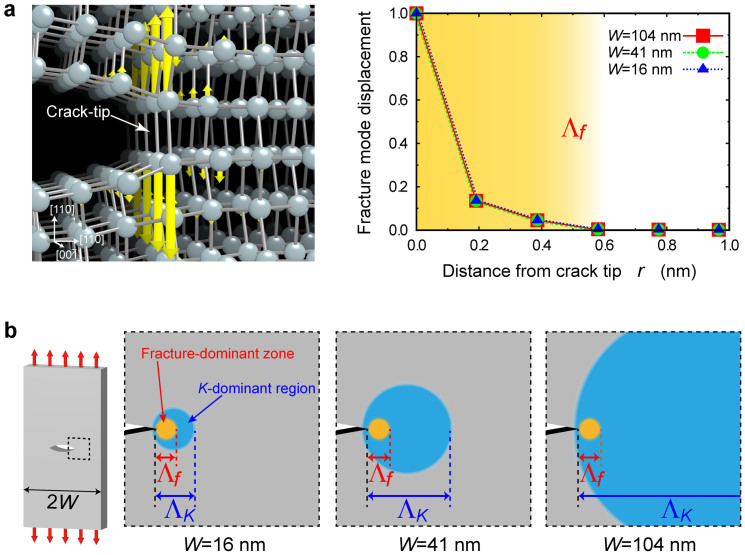
Brittle fracture mode at the crack-tip. (a), Visualization of fracture mode at the onset of brittle fracture (left panel), and the fracture mode displacement normalized by the maximum displacement at the crack-tip for the different size of specimens (right panel). Λ*_f_* denotes the fracture-dominant zone. The fracture mode is analysed by instability mode analysis (see supplementary information). (b), *K*-dominant region Λ*_K_* vs. fracture-dominant zone Λ*_f_* in different size of specimens. Λ*_K_/*Λ*_f_* ≈ 10, 4, and 2 for the specimens of *W* = 104, 41, and 16 nm, respectively.

**Figure 4:**
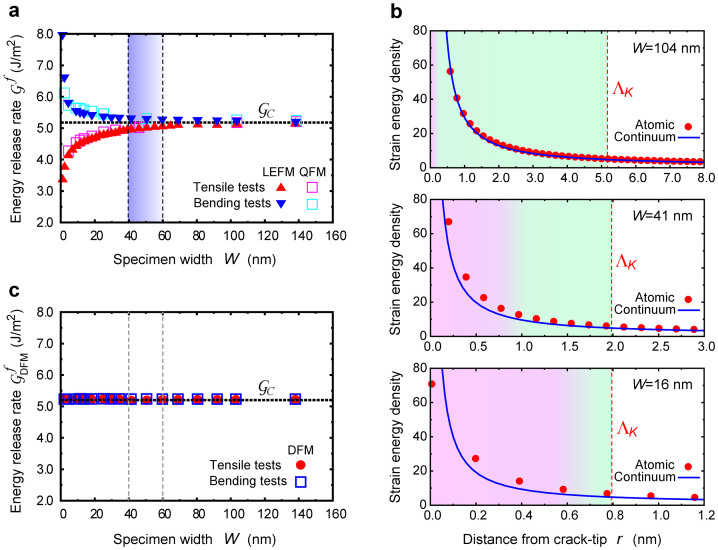
Energy-based criteria on the basis of continuum vs. discrete fracture mechanics. (a), Critical energy release rate at fracture 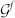 based on linear elastic fracture mechanics (LEFM) and quantized fracture mechanics (QFM) as a function of specimen size *W*. The LEFM and QFM are both based on the conventional continuum mechanics. The horizontal dotted lines indicate the fracture toughness 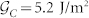. (b), Comparison of strain energy density distributions near the crack-tip between the actual atomic specimen and the continuum assumption in three different size of specimens under the critical loading conditions. The strain energy density is normalized by the averaged strain energy density of the entire specimen. In the large (104 nm) specimen where fracture mechanics works the continuum strain energy is in good agreement with the actual one, while the continuum strain energy dramatically deviates from the actual one in critically small specimens (41 and 16 nm). (c), Critical energy release rate at fracture 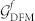 based on discrete fracture mechanics (DFM) proposed here as a function of specimen size *W*.
