# Supplementary material for: Breakdown of Continuum Fracture Mechanics at the Nanoscale
Source: Sci Rep. 2015 Feb 26;5:8596. doi: 10.1038/srep08596 (PMC4341196; doi:10.1038/srep08596)
Supplement: Supplementary Information — Supplementary Figures and Theory [file srep08596-s1.pdf]

Supplementary Information

-Breakdown of continuum fracture mechanics at the nanoscale-

Takahiro Shimada,[1,*] Kenji Ouchi,[1] Yuu Chihara,[1] and Takayuki Kitamura[1]

[1] *Department of Mechanical Engineering and Science, Kyoto University, Nishikyo-ku, Kyoto 615-8540, Japan*

Instability Mode Analysis: A theory of mechanical instabilities in arbitrary atomic systems[1,2]

Let us consider an arbitrary atomic system consisting of N atoms. The potential energy of the atomic system, U, is a functional of atomic coordinate, and is given by

$$U = U(\boldsymbol{R}), \tag{1}$$

where $\boldsymbol{R}$ denotes the configuration vector consisting of atomic positions,

$$\boldsymbol{R} \equiv (r_x^{(1)}, r_y^{(1)}, r_z^{(1)}, r_x^{(2)}, r_y^{(2)}, r_z^{(2)}, \ldots, r_i^{(\alpha)}, \ldots, r_x^{(N)}, r_y^{(N)}, r_z^{(N)}). \tag{2}$$

Here, $r_i^{(\alpha)}$ denotes the coordinate of atom α in the i direction. This is the general form to express the potential energy of atomic system, in any type of potential functions and force field including *ab initio* method. The irreducible number of degrees of freedom (DOFs) in the atomic system without any constraint is $M = 3N-6$ because the DOFs of rigid-body translation (3) and rotation (3) is subtracted from the total DOFs of atoms ($3N$). Under a displacement constraint where some of atoms are fixed, the number of DOFs is $M=3N-3n_c-6$, where n_c is the number of constrained atoms. Here, let us consider the atomic system under static external load, i.e., the atoms are located at their own optimal sites and are in balance with the external load and/or constraint. The total energy Π of the equilibrated atomic configuration ($\boldsymbol{R}_0$) under external load now consists of the

* E-mail: shimada@me.kyoto-u.ac.jp

potential energy U and the work done by external load W, and is given by

$$\Pi = U + W . \tag{3}$$

Assuming an infinitesimal deformation $\delta\boldsymbol{R}$ to the equilibrated system, the total energy of slightly deformed system $\Pi(\boldsymbol{R}_0 + \delta\boldsymbol{R})$ can be described by the Taylor's series expansion of total energy $\Pi(\boldsymbol{R}_0)$ with respect to $\delta\boldsymbol{R}$, and is given by

$$\Pi(\boldsymbol{R}_0 + \delta\boldsymbol{R}) = \Pi(\boldsymbol{R}_0) + \sum_{m=1}^{M} \left(\left.\frac{\partial U}{\partial R_m}\right|_{\mathbf{R}=\mathbf{R}_0} + \left.\frac{\partial W}{\partial R_m}\right|_{\mathbf{R}=\mathbf{R}_0} \right) \delta R_m$$
$$+ \frac{1}{2}\sum_{m=1}^{M}\sum_{n=1}^{M} \left.\frac{\partial^2 U}{\partial R_m \partial R_n}\right|_{\mathbf{R}=\mathbf{R}_0} \delta R_m \delta R_n + \cdots + \frac{1}{2}\sum_{m=1}^{M}\sum_{n=1}^{M} \left.\frac{\partial^2 W}{\partial R_m \partial R_n}\right|_{\mathbf{R}=\mathbf{R}_0} \delta R_m \delta R_n + \ldots, \tag{4}$$

where R_m denotes a component of configuration vector $\boldsymbol{R}$ included in the DOFs of system, i.e., $R_m = r_i^{(\alpha)}$. Since the first derivative of total energy (i.e., force acting on atoms) is zero due to the system at equilibrium, the second term on the right-hand side can be eliminated.

$$\frac{\partial \Pi}{\partial R_m}\delta R_m = \left(\left.\frac{\partial U}{\partial R_m}\right|_{\mathbf{R}=\mathbf{R}_0} + \left.\frac{\partial W}{\partial R_m}\right|_{\mathbf{R}=\mathbf{R}_0} \right)\delta R_m = 0 \quad (m=1,\ \ldots,\ M) \tag{5}$$

Considering that the external load is constant due to the static loading, the work is proportional to the displacement of atoms on which the external load is applied. Thus, we get

$$\frac{\partial^2 W}{\partial R_m \partial R_n}\delta R_m \delta R_n = 0 \qquad (m,n=1,\ \ldots,\ M). \tag{6}$$

Using Eqs. (4)-(6) and ignoring the higher-order terms, the total energy change $\delta\Pi$ with respect to the infinitesimal deformation $\delta\boldsymbol{R}$ is given by

$$\delta\Pi(\boldsymbol{R}_0) = \Pi(\boldsymbol{R}_0 + \delta\boldsymbol{R}) - \Pi(\boldsymbol{R}_0) = \frac{1}{2}\sum_{m=1}^{M}\sum_{n=1}^{M}\frac{\partial^2 U}{\partial R_m \partial R_n}\delta R_m \delta R_n = \frac{1}{2}\delta\boldsymbol{R}^{\mathrm{T}}\mathbf{H}\delta\boldsymbol{R}, \tag{7}$$

where $\mathbf{H}$ is the $M \times M$ Hessian matrix of potential energy Π with respect to $\mathbf{R}$ and ^{T} means transposition. The component of Hessian matrix, H_{ij}, is

$$H_{mn} \equiv \left.\frac{\partial^2 U}{\partial R_m \partial R_n}\right|_{\mathbf{R}=\mathbf{R}_0} \qquad (m,n=1,\ \ldots,\ M). \tag{8}$$

By solving the eigenvalue problem of the Hessian matrix $\mathbf{H}$,

$$\mathbf{H}\boldsymbol{p}_m = \eta_m \boldsymbol{p}_m, \tag{9}$$

where η_m ($\eta_1 \le \ldots \le \eta_m \le \ldots \le \eta_M$) is the eigenvalue of the Hessian matrix $\mathbf{H}$, and $\boldsymbol{p}_m$ is the corresponding eigenvector. Using the eigenvector $\boldsymbol{p}_m$, the Hessian matrix is diagonalized as

$$\mathbf{P}^{-1}\mathbf{HP} = \mathbf{P}^{\mathrm{T}}\mathbf{HP} = \begin{pmatrix} \eta_1 & & O \\ & \ddots & \\ O & & \eta_M \end{pmatrix}, \tag{10}$$

where $\mathbf{P} = (\boldsymbol{p}_1 \ldots \boldsymbol{p}_M)$. Since the eigenvectors are an orthogonal basis set of M-dimensional vector space, any infinitesimal deformation $\delta\mathbf{R}$ can be expressed as a linear combination of the eigenvectors as

$$\delta\boldsymbol{R} = \sum_{m=1}^{M} u_m \boldsymbol{p}_m = \mathbf{P}\boldsymbol{u}, \tag{11}$$

where u_m is the component of $\delta\mathbf{R}$ in the $\boldsymbol{p}_m$ direction and $\boldsymbol{u} = (u_1, \ldots, u_M)$. Therefore, the total energy change by $\delta\mathbf{R}$ in Eq. (7) becomes

$$\delta\Pi(\boldsymbol{R}_0) = \frac{1}{2}(\mathbf{P}\boldsymbol{u})^{\mathrm{T}}\mathbf{H}(\mathbf{P}\boldsymbol{u}) = \frac{1}{2}\boldsymbol{u}(\mathbf{P}^{\mathrm{T}}\mathbf{HP})\boldsymbol{u} = \frac{1}{2}\sum_{m=1}^{M}\eta_m u_m^2 . \tag{12}$$

When the minimum eigenvalue is positive, $\eta_1 > 0$ (i.e., all the eigenvalues are positive), the atomic system is confirmed to be mechanical stable because $\delta\Pi$ is always positive with respect to any infinitesimal deformation $\delta\mathbf{R}$. In contrast, when the minimum eigenvalue is negative, $\delta\Pi$ becomes negative with respect to $\delta\mathbf{R} = \boldsymbol{p}_1$, indicating that the atomic system is mechanical unstable along the deformation path of $\delta\mathbf{R} = \boldsymbol{p}_1$. Therefore, the mechanical stability/instability of given atomic system can be determined by the sign of minimum eigenvalue η_1 of Hessian matrix, and the deformation mode at the onset of mechanical instability can be identified by the

corresponding eigenvector $\boldsymbol{p}_1$. Since the cleavage fracture focused in the present study accompanies bond breaking at the crack-tip, this is clearly one of intrinsic mechanical instabilities in a brittle atomic system, and the atomistic and discrete nature of brittle fracture at the crack-tip can be described by eigenvector $\boldsymbol{p}_1$ from our approach, as shown in Fig. 3a and Supplementary Fig. S2.

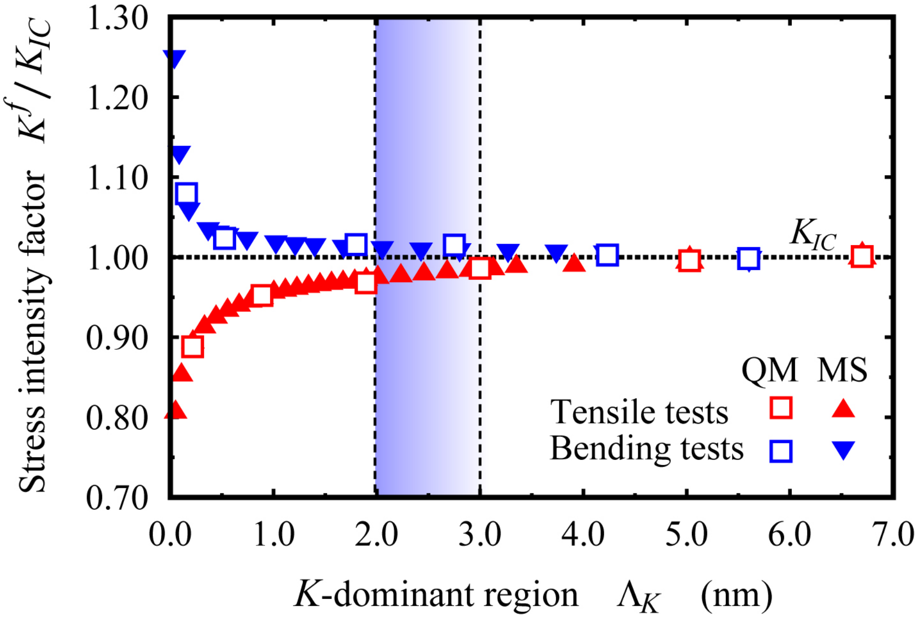

Supplementary Figure S1 | Critical stress intensity factors at fracture obtained from fracture tests based on the quantum-mechanics (QM) vs. classical molecular statics (MS) simulations. The critical stress intensity factor at fracture K_I^f is evaluated from the continuum stress distribution near the Si(110) crack-tip at a critical tensile load or bending moment obtained by the QM-based computational fracture experiments using the first-principles density-functional theory (DFT) calculations within the generalized gradient approximation, and the MS-based fracture tests using the bond-order potential for brittle fracture of silicon. For the detailed theory and models of QM simulations, see the method section and supplementary Fig. S3.

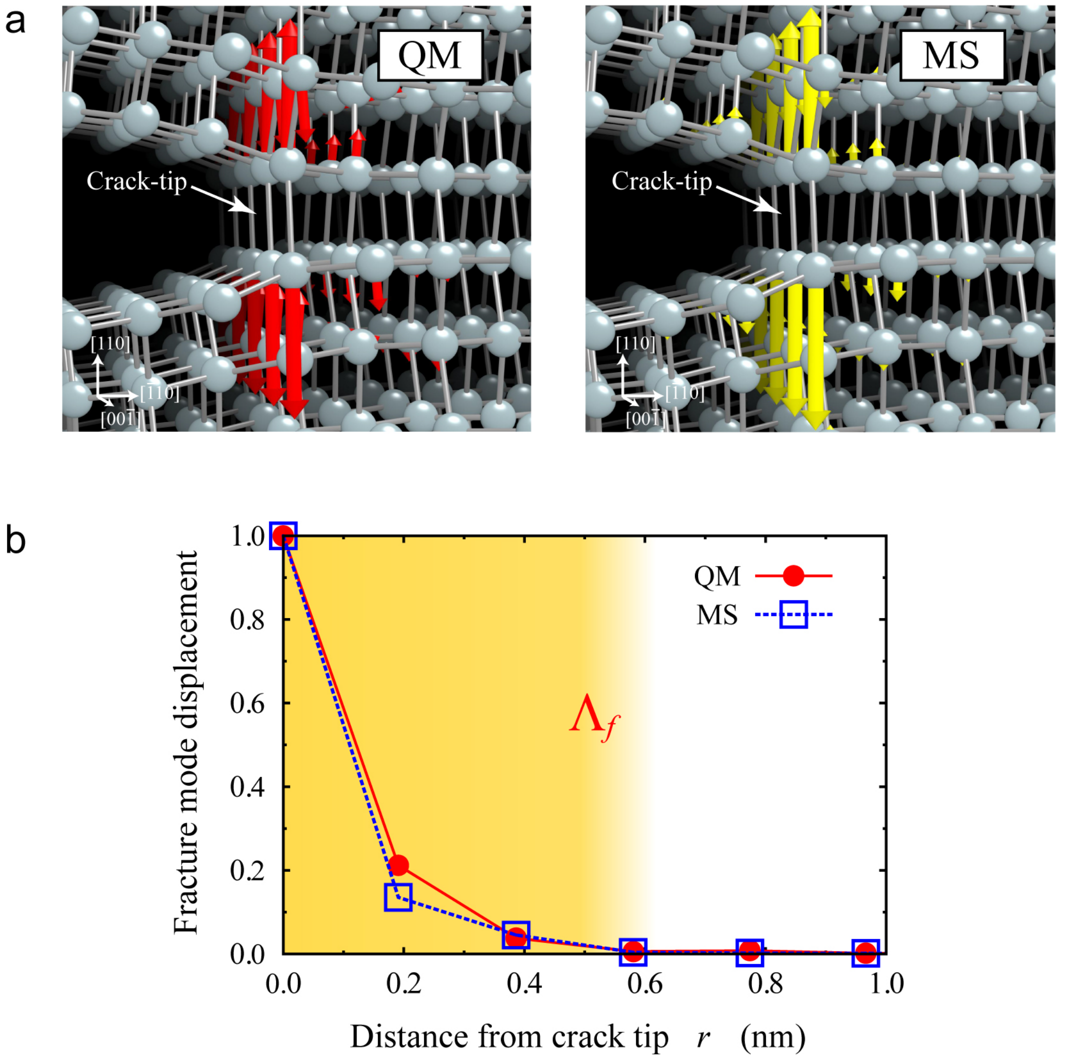

Supplementary Figure S2 | Comparison of fracture mode displacement obtained by quantum-mechanics (QM) and classical molecular statics (MS) simulations. The fracture mode displacement obtained by our instability mode analysis, the details of which are described in this Supplementary Information above. The quantum-mechanics results are based on the first-principles density-functional theory (DFT) calculations within the generalized gradient approximation. For the detailed theory and models, see the method section and Supplementary Fig. S3.

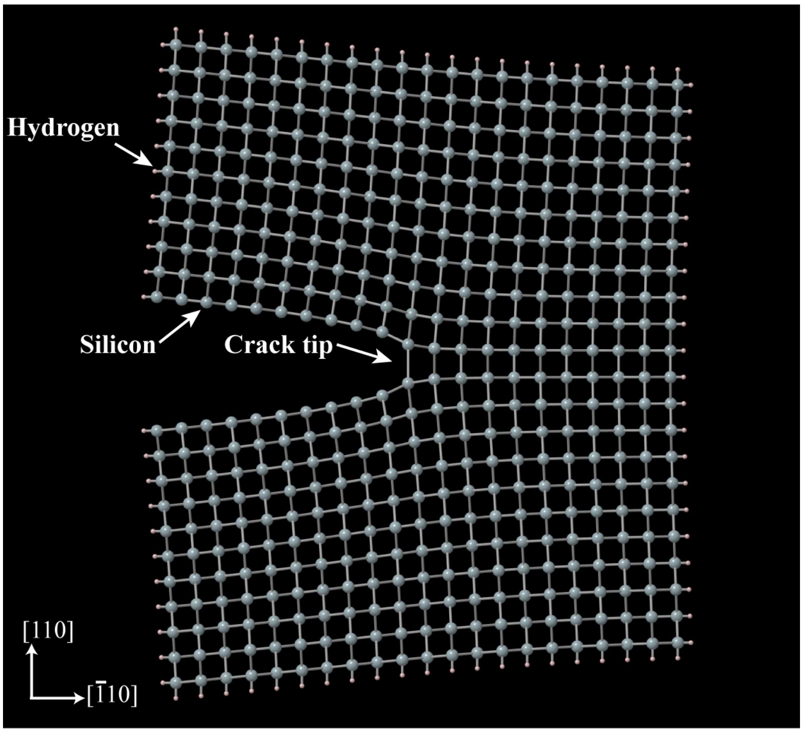

Supplementary Figure S3 | Simulation model of the (110) crack in silicon for quantum-mechanics (QM) density-functional theory calculations. Si atoms at model surfaces are passivated by H-termination. The model consists of 462 Si atoms and 86 H atoms. The position and displacement of Si atoms at the two atomic layers from the surface are controlled in order to feedback the elastic strain field outside the model. The present models and procedure is previously validated.[3]

References

1. Kitamura, T., Umeno, Y. & Fushino, R. Instability criterion of inhomogeneous atomic system. *Mater. Sci. Eng. A* **379**, 229-233 (2004).

2. Shimada, T., Okawa, S., Minami, S. & Kitamura, T. Simplified evaluation of mechanical instability in large-scale atomic structures. *Mater. Sci. Eng. A* **513-514**, 166-171 (2009).

3. Pérez, R. & Gumbsch, P. An ab initio study of the cleavage anisotropy in silicon. *Acta Mater.* **48**, 4517-4530 (2000).
